# Diphtheria

**Published:** 1861-01

**Authors:** T. J. Pearce

**Affiliations:** Decatur, Indiana


### DIPHTHERIA.

By T. J. PEARCE, M. D., of Decatur, Indiana.

*Editors Examiner.*—The late fearful malady known as *Diphtheria*, having prevailed in our vicinity more or less for the last two years, and having in its ravages taken as one of its victims my last and only son, and very nearly destroyed our only remaining child, a little girl of five years old, I will briefly give you the result of my observation as to its general character, and most appropriate treatment.

1st. I think Diphtheria belongs nosologically to the family of Scarlatina, and as such, consists pathologically in a blood change.

My experience teaches me that it is not contagious ; and yet in certain conditions it may be propogated by contagion.

I am satisfied my little daughter took it without any contagious bearing in the case, and I am equally certain that my son took it by contagion from her.

The Diphtheritic deposit or adventitious membrane so generally present in some part of the faucal structure, I view as belonging in its formation to the healthiest pathological stage of the disease; especially so in the malignant variety of Diphtheria. As when the blood change passes the point of this *plastic deposit*, the cases are generally hopeless, and this brings us to notice the outlines of the treatment. And here I would say that I think we are prone to look too much to the *effects* of the disease, the throat affection, and not pay enough attention to the disease itself, the blood change. I think the too frequent *swabbing*, has been in many cases a fruitful source of mischief. By this, the nervous little sufferers are not only thrown into frequent spells of the greatest bodily and mental excitement, which in all blood diseases should be avoided, but the tender and swollen parts are farther irritated and made worse instead of better. And yet we would have the parts *cleansed* of the foul secretions by the moderate use of gargles, or the *probang*, where it can be done without too much risk of increasing the general and local difficulty. But our principal reliance is in the full and free use of the constitutional remedies. And to fill this indication we know of no course better than the free use of Chlorate of Potash, Mur. Tinct. Ferii. and Sul. Quinia. The Mur. Tinct. Ferri. I think is generally given too sparingly. As a combined local and general agent, I would call attention to Sul. Cupri. as an emetic, whenever it is administered in the course of the disease, also as a gargle and an internal agent in small doses. When given as an emetic, we not only get its prompt effects in that way, but it acts as a valuable local application in passing thoroughly over the mucous membrane of the fauces and pharynx. And in the croupal form (which I consider strictly, not a *variety*, but an incidental condition) of the disease, I think there is no remedy which promises more success than Sul. Ferri. as an emetic. The various remedies used as local applications, Nit. Silver, Tinct. Iodine, Tannic Acid, Capsicum, Sul. Zinc, *Gunpowder*,

Alum, etc., each may doubtless do good in its *time* and *place*, but I think their frequent use with the sponge, may often do much harm. As an external application when there is edema, or enlargement of the parotid or cervical glands, I have found nothing better than the free use of Tinct Iodine. A domestic poultice of equal parts of *Tar* and wheat bran may ofton prove servicable.

But I look upon *Malignant Diphtheria*, when it, in the blood degeneration, has passed the point of forming the *organized deposit*, and becomes clothed with the usual symptoms that then attend it, as necessarily fatal, and to ease the little sufferer's passport from time into eternity, as the highest duty we can then perform.

